# Integrating Carbon Nanomaterials with Metals for Bio-sensing Applications

**DOI:** 10.1007/s12035-019-01767-7

**Published:** 2019-09-14

**Authors:** Sami Sainio, Elli Leppänen, Elsi Mynttinen, Tommi Palomäki, Niklas Wester, Jarkko Etula, Noora Isoaho, Emilia Peltola, Jessica Koehne, M. Meyyappan, Jari Koskinen, Tomi Laurila

**Affiliations:** 1 Stanford Synchrotron Radiation Lightsource, SLAC National Accelerator Laboratory, Menlo Park, CA 94025 USA; 2 Department of Chemistry and Materials Science, School of Chemical Technology, Aalto University, 02150 Espoo, Finland; 3 Department of Electrical Engineering and Automation, School of Electrical Engineering, Aalto University, 02150 Espoo, Finland; 4 Center for Nanotechnology, NASA Ames Research Center, Moffett Field, Mountain View, CA 94035 USA

**Keywords:** Carbon, Carbon nanomaterials, Bio-sensing, Dopamine

## Abstract

Age structure in most developed countries is changing fast as the average lifespan is increasing significantly, calling for solutions to provide improved treatments for age-related neurological diseases and disorders. In order to address these problems, a reliable way of recording information about neurotransmitters from in vitro and in vivo applications is needed to better understand neurological diseases and disorders as well as currently used treatments. Likewise, recent developments in medicine, especially with the opioid crisis, are demanding a swift move to personalized medicine to administer patient needs rather than population-wide averages. In order to enable the so-called personalized medicine, it is necessary to be able to do measurements in vivo and in real time. These actions require sensitive and selective detection of different analytes from very demanding environments. Current state-of-the-art materials are unable to provide sensitive and selective detection of neurotransmitters as well as the required time resolution needed for drug molecules at a reasonable cost. To meet these challenges, we have utilized different metals to grow carbon nanomaterials and applied them for sensing applications showing that there are clear differences in their electrochemical properties based on the selected catalyst metal. Additionally, we have combined atomistic simulations to support optimizing materials for experiments and to gain further understanding of the atomistic level reactions between different analytes and the sensor surface. With carbon nanostructures grown from Ni and Al + Co + Fe hybrid, we can detect dopamine, ascorbic acid, and uric acid simultaneously. On the other hand, nanostructures grown from platinum provide a feasible platform for detection of H_2_O_2_ making them suitable candidates for enzymatic biosensors for detection of glutamate, for example. Tetrahedral amorphous carbon electrodes have an ability to detect morphine, paracetamol, tramadol, and *O*-desmethyltramadol. With carbon nanomaterial-based sensors, it is possible to reach metal-like properties in sensing applications using only a fraction of the metal as seed for the material growth. We have also seen that by using nanodiamonds as growth catalyst for carbon nanofibers, it is not possible to detect dopamine and ascorbic acid simultaneously, although the morphology of the resulting nanofibers is similar to the ones grown using Ni. This further indicates the importance of the metal selection for specific applications. However, Ni as a continuous layer or as separate islands does not provide adequate performance. Thus, it appears that metal nanoparticles combined with fiber-like morphology are needed for optimized sensor performance for neurotransmitter detection. This opens up a new research approach of application-specific nanomaterials, where carefully selected metals are integrated with carbon nanomaterials to match the needs of the sensing application in question.

## Introduction

Based on both European and US sources [1, 2], more than a quarter of the population suffers from different neurological disorders. These figures are expected to increase as the population’s age structure is changing with the increasing average lifespan. In addition to the above, the current opioid crisis raises questions if the population-wide averages for deciding the dosing for pain medication have been the right approach. Both problems could be solved by realization of personalized medicine, i.e., the ability to quickly, reliably, and affordably record how each of the patients responds to given treatment. For example, recording analgesic-related metabolites from blood would allow determining individual response to the administered medicine for each patient.

There is an abundant number of publications on the detection of neurotransmitters using different carbon nanomaterials (CNM) and their composites, some of which are listed in Table 1 and in a review by Meyyappan [3]. However, as pointed out in a recent critical review [4], the connection between the sensing/electrochemical performance and the physicochemical properties of the sensing material has not been established unambiguously. The understanding of the effect of carbon, its functional groups (O, N), and the residing metal impurities from the fabrication process have recently gained more attention, but the scientific community has yet to determine what truly enables the electrocatalytic properties of the CNMs. In addition to the complex carbon-metal composite structures resulting from the growth process of these carbon nanostructures, their morphology is reported to have a significant role in the performance.

**Table 1:** Collection of materials sensitive towards DA and capable of selective detection of DA and AA

Electrode material	Seed material/metal catalyst	Lowest measured DA concentration (nM)	Method	Ref.
50 nm ta-C + MWCNT	Al + Co + Fe	50	CV	Palomäki et al. 2018
15 nm ta-C + MWCNT	Al + Co + Fe	10	CV	Palomäki et al. 2018
7 nm ta-C + CNT	Al + Co + Fe	500	CV	Sainio et al. 2015a
7 nm ta-C + CNF	Ni	500	CV	Sainio et al. 2015b
CNF	Ni	50	DPV	Rand et al. 2013
CNS	Pt	1000	CV	Wang et al. 2011
GC + MWCNT	Pt	5000	CV	Dursun and Gelmez 2010
		61	DPV	Dursun and Gelmez 2010
MWCNT	Ta	10,000	CV	Poh and Loh 2004
N-MWCNT	C_10_H_10_Fe	120	CV	Tsierkezos et al. 2016

*CNS* carbon nanosheet, *MWCNT* multi-wall carbon nanotube

CNMs fabricated from different metal seed layers can result in similar micro- and nanoscale structures, but have clearly different electrocatalytic properties. Here, we show a collection of results from recent publications from our group and correlate them with new studies of CNM grown without metallic seed layer, and the same samples with additional Ni catalyst layer, with (tetrahedral amorphous carbon) ta-C + nanodiamonds (ND), with ta-C + Fe seed hybrid and with ta-C + 2 nm Ni film. We have found that the use of Al-Co-Fe hybrid seed layer for CNM growth yields integrated structures that can detect dopamine (DA), ascorbic acid (AA), and uric acid (UA) sensitively and selectively (allowing simultaneous detection of the analytes) [5]. Further, using only Fe as seed for growing carbon nanofibers (CNF) allows us to selectively detect DA and AA, but the sensitivity is poor. Pt-grown CNFs are insensitive for DA and are passivated in just several measurement cycles. However, with both Pt- and Ni-grown CNF, we can detect H_2_O_2_ arising from the enzymatic reactions of glutamate [6]. From these two, the Pt version is much more sensitive towards H_2_O_2_ [6]. The detection of neurotransmitters and H_2_O_2_ is not feasible with morphologically similar CNF that do not have any catalytic metals in large quantities (≥ 0.1 at. %).

We have utilized single-wall carbon nanotubes (SWCNT) with Fe seed and ta-C films with very low amount of metals (< 0.1 at.%) to successfully detect morphine (MO) and paracetamol (PA) [7], as well as tramadol (TR) and *O*-desmethyltramadol (ODMT) [8]. In the application of TR and ODMT using ta-C as sensor surface, the ability to detect these analytes is based on the very wide (nearly 4 V) water window of ta-C. [7, 8].

Thus, to understand what is causing the electrocatalytic properties of the CNM, it is important to study the effects of (i) morphology (feature length, diameter, and size), (ii) carbon structure and orientation (crystalline/amorphous, basal/edge plane, and amount of defects), (iii) surface chemistry and functionalization, (iv) alloying, and (v) nature and abundance of metals.

It is practically impossible to understand the root causes behind the observed complex performance solely on the basis of the heavily convoluted experimental data, which cannot provide any atomistic level information about our systems. Thus, computational methods augmented by machine learning techniques are required (i) to deconvolute the experimental results into atomic level information and (ii) to aid in developing an atomic-scale quantitative microscopic model for various interactions occurring in the system. We have recently combined computational methods, experimental work, and machine learning techniques to deconvolute X-ray absorption data into atomic-scale surface chemical information by utilizing the so-called fingerprint spectra [9–11]. This atomic-scale chemical information can then be compared with the observed electrochemical behavior to reveal the fundamental connections between surface chemistry and electrocatalysis. This information then gives us the possibility to tailor the carbon surface chemistry for a specific application. Likewise, we have utilized density functional-based methods to investigate atomic level interactions between different catalyst metals and carbon to rationalize the different morphologies observed.

The above machine learning augmented by computational approach could also prove to be extremely useful for interpretation and rationalization of electrochemical data, since many of the recorded voltammograms contain heavily overlapping peaks. However, despite the main peaks overlapping, it is likely that if the whole voltammogram of a given analyte (its fingerprint) is “learned,” features that are unique for a given analyte can be derived. Thus, by learning these fingerprints of different analyte reactions on given electrode materials, it could be possible to enable the required selectivity by machine learning-induced peak deconvolution.

## Experimental

CNMs utilizing metal seed layers were grown using either chemical vapor deposition (CVD) or plasma-enhanced CVD (PECVD) process at temperatures of 550 to 750 °C. The processes are explained in detail in our previous publications [12–14]. Briefly, the hybrid Al-Co-Fe seed layered material was deposited on top of a 15- and 50-nm ta-C containing Si wafer using an electron beam [5] aiming for 0.2 nm of Al, 2 nm of Fe, and 2 nm of Co. After deposition, the samples were taken to Black Magic CVD reactor (Aixtron, Germany) and heated up to 550 °C for 10 min with NH_3_ flow at 250 sccm [12]. Samples with 20 nm Ni, 20 nm Fe, and 10 nm Pt seed layers were deposited on top of 7 nm ta-C film and grown in Black Magic PECVD (Aixtron, Germany) chamber at 750 °C for 30 min with C_2_H_2_ flow of 25 sccm and NH_3_ flow of 125 sccm. The ND-grown carbon nanofibers were also fabricated using the PECVD reactor mentioned above. The carboxylated nanodiamonds were deposited on the initial sample surface by drop-casting, where commercially available aqueous diamond solution with a concentration of 5 wt% (Vox, Carbodeon, Finland) was diluted to a concentration of 0.05 wt% with deionized water. A 40-μL drop was then drop-casted on the sample surface, placed on a glass slide. Finally, the samples were dried on a preheated hot plate at 85 °C for 10 min to accelerate the evaporation of solvent. The ND used here are commercially available detonation ND with brand name Vox by Carbodeon for CNF growth (carboxyl functionalized) and Andante (oxygen functionalized surface with non-specific groups) for ta-C + ND and for the ND + 20 nm Ni CNF growth.

Cyclic voltammetry (CV) measurements were carried out using Gamry Reference 600 or 600+ potentiostat. A three-electrode setup was used with an Ag/AgCl reference electrode (Radiometer Analytica) and a Pt wire (Goodfellow) as a counter electrode. Dopamine hydrochloride, l-ascorbic acid, and uric acid were purchased from Sigma-Aldrich. Fresh solutions of DA, AA, and UA were prepared on the day of the measurements. Phosphate buffer saline (PBS) with pH 7.4 was used as an electrolyte. All solutions were purged with nitrogen for 30 min before and blanketed during the experiments. All measurements were conducted at room temperature in a Faraday cage. The geometric area of the Al + Co + Fe-grown electrode was 0.071 cm^2^ whereas for rest of the electrode area was 0.031 cm^2^.

High-resolution transmission electron microscopy (HRTEM) studies were carried out using JEOL JEM-2200FS under 200 kV. Samples were prepared by EAG Laboratories (USA) using focused ion beam (FIB) thinning of an extracted lamella. Scanning electron microscopy (SEM) was carried out with JEOL and Hitachi S-4700 scanning electron microscopes. Energy-dispersive X-ray spectroscopy (EDS) was carried out at low magnification using Tescan MIRA3 scanning electron microscope equipped with Thermo Scientific UltraDry silicon drift X-ray detector. Five spots approximately 1 × 1 mm in size were analyzed for each sample.

## Results

**Fig. 1:**
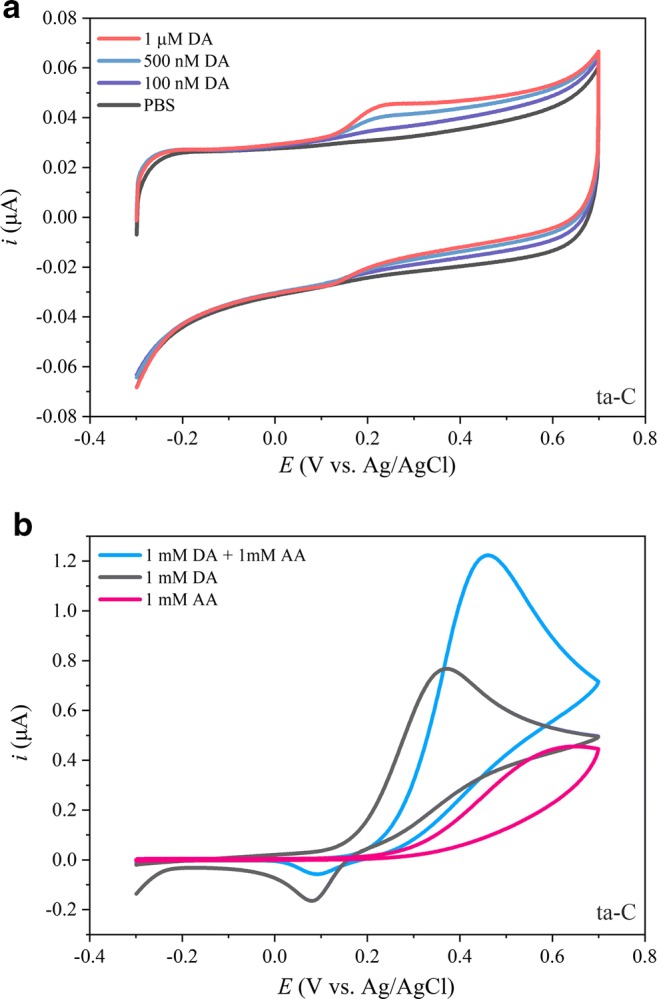
Electrochemical performance of the Si + 20 nm Ti + 7 nm ta-C thin film is shown in panels **a** and **b**. Scan rate 50 mV/S for all measurements is shown [5]

In the analysis and discussion of the physicochemical results from the new carbonaceous nanomaterials, we will utilize our most well-studied material as the reference, a 7-nm-thick ta-C layer deposited on 20-nm Ti adhesion layer deposited on Si wafer. The fabrication process, properties of the resulting film, and its electrochemical performance have been described and discussed in considerable detail in our previous publications [5, 12]. Electrochemical performance of the ta-C films towards DA and AA is shown in Fig. 1 for reference and there is both the lack of sensitivity and selectivity when only one carbon allotrope is utilized.

Both physiologically meaningful sensitivity and selectivity must be achieved to evaluate the material’s applicability for neurotransmitter detection. This means concentrations as low as 5–700 nM for DA [15, 16] and clear peak separation in the voltammogram against (at least) AA and UA (see Fig. 3d and f for proper peak separation). Additionally, it is necessary to use CV measurements instead of differential pulse voltammetry (DPV) measurements to capture real-time data.

**Fig. 2:**
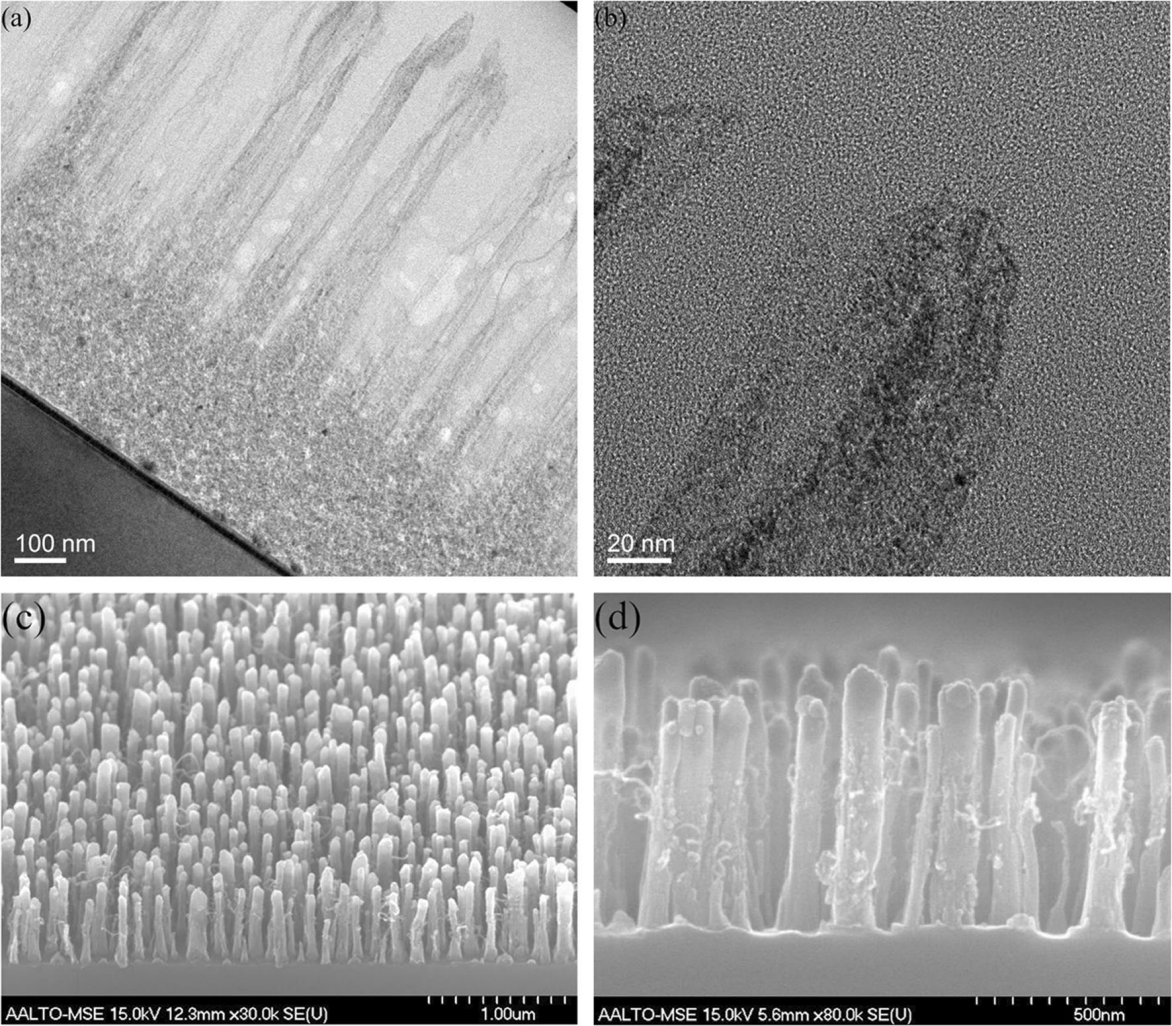
**a**, **b** TEM micrographs of ND-grown CNF where in panel **a**, the 700-nm-tall fibers are grown on top of a ND layer, in panel **b**, the tip of the resulting fiber is shown and in panel **c**, body of the fiber is shown that shows amorphous areas where the diamonds are scattered around the body of the fiber. **c**, **d** SEM micrographs of the Fe-grown CNF showing similar morphology as the fibers in refs [17–19]

As Fig. 1a shows, the sensitivity of the ta-C film towards DA is nearly adequate at 500 nM–1 μM but that the peak separation between DA and AA in panel b is too small to selectively detect them as separate peaks.

### Structural Characterization

In order to induce the required sensitivity and especially selectivity, we have utilized the so-called integrated carbon nanomaterials where different carbon allotropes are integrated together with the aid of catalyst materials, which are typically metals. We present the results from these studies below and try to find common roots that could be identified behind the increased sensitivity and selectivity. We will start by considering the metal-free carbon nanofibers where nanodiamonds have been used as a catalyst layer instead of the typical metals.

Comparing the HRTEM micrographs from the ND-grown CNF shown in Fig. 2a–c, it is evident, that the morphology of the resulting fibers is similar to earlier results [14, 17] where 700-nm-long and tens to hundreds of nm wide fibers have resulted from Ni [18], Fe (as shown in SEM micrographs in Fig. 2), and Pt [19] seeds under similar PECVD conditions. The main differences are that the ND-grown fibers lack the metallic tip found from the metal seed grown fibers, as expected. The visible darker-colored grains in the ND-grown fiber walls and body are titanium that was used as an adhesion layer for the ta-C that was deposited under the ND film. Additionally, there is a difference in the carbon forming the fibers. The ND-grown fibers seem to have mainly amorphous carbon (a-C) structure, the Ni, Fe, and Pt-grown fibers have more clear ordered structures, but even the Ni and Pt fibers are different in their structure which will be discussed in more detail later.

### Electrochemical Detection of Neurotransmitters

**Fig. 3:**
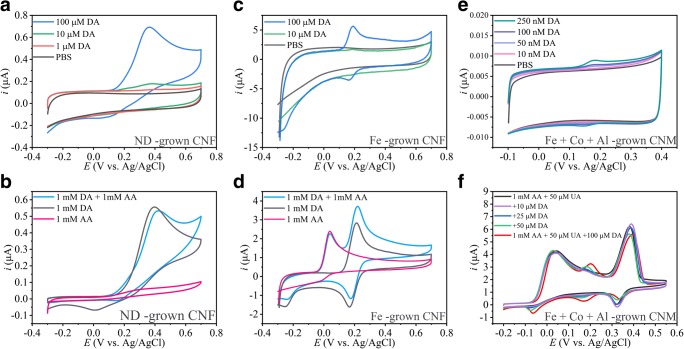
Cyclic voltammograms from measurements with ND, Fe, and Fe + Co + Al-grown CNM. In panels **a** and **b**, the ND-grown CNF shows no detection for AA and low sensitivity towards DA. In panels **c** and **d**, Fe-grown CNF show selectivity of DA and AA but low sensitivity to DA and in panels **e** and **f**, the best results so far for both sensitivity and selectivity are shown with Fe + Co + Al-grown CNM. Scan rate of 50 mV/s is shown for all measurements

Electrochemical measurements for the metal-free ND-grown CNF, Fe-grown CNF, and Al + Co + Fe-grown CNM are shown in Fig. 3 a and b, c and d, and e and f, respectively. Figure 3 clearly shows that neither the required sensitivity or selectivity is achieved with ND-grown CNF and only selectivity is achieved with Fe-grown CNF. This indicates that ND-grown CNF, although having very similar morphology as for example the Ni-grown CNF, is not viable for neurotransmitter detection. It should be noted, that although both fiber macroscopic structure appears to be similar, fiber-like, but microscopic structure differs as the ND-grown CNF are mainly a-C, whereas the Ni-grown CNF are crystalline. Interestingly, both sensitivity and selectivity over DA for both AA and UA were achieved with Al + Co + Fe-grown CNM. This is proposed to arise from a mixture of properties: (i) there are at least Fe and Co metal nanoparticles available, (ii) there are crystalline carbon areas available, and (iii) the morphology in both micro- and macroscale is optimal for electrocatalysis (high surface area and the CNM forms a porous network that enables enrichment of the analyte).

The only material that we have found to show selective behavior towards DA and AA without growth seed metal particles is ta-C + partially reduced graphene oxide (PRGO) hybrid electrode that has been treated with concentrated nitric acid before the measurements [20]. Based on the X-ray photoelectron spectroscopy (XPS) studies, the surface of the PRGO is covered by more than 30 at.% oxygen, more than 10 at.% Si, and traces of at least S, N, B, Ca, and possible traces of Al, F, Mn, and Na (it should be noted that KMnO_4_ is used in the process of making the PRGO and the Mn is not removed from the material by any specific process and is likely present in higher concentration in the bulk of the PRGO). Due to the complexity of the PRGO surface, it is hard to attribute the selective behavior to any single component of the system. However, we believe that the enhanced performance is due to the heavily oxidized system, increased surface area due to the formation of a porous membrane-like structure, and to the Mn present in the system.

Table 1 shows the results for different materials’ capability for DA and AA separation, the electrochemical method used, and the seed material used for the CNM growth. Only materials with capabilities for simultaneous DA and AA detection are shown.

As shown in Table 1, the only materials we have found to fulfill the requirements of sensitivity and selectivity are the hybrid Fe + Co + Al-grown CNM and the Ni-grown CNF. Other results listed in Table 1 show that simultaneous detection of DA and AA is possible with CNM grown from Pt, Ta, and Fe. Based on our knowledge, the Fe + Co + Al-grown CNM is the only material capable of detecting DA, AA, and UA sensitively and selectively with CV [5], whereas the Ni-grown CNF has shown adequate performance only with DPV [21]. Results with DPV are not relevant due to the lack of application required time resolution (in the case of neurotransmitter detection).

Some materials, such as the CNF grown using Fe seed, show clear peak separation for DA and AA (see Fig. 3) but lack the necessary sensitivity. Other materials without metal particles, like the ta-C films, show good enough sensitivity but lack the selectivity. Furthermore, by building carbon hybrid nanomaterials, e.g., by combining ta-C and ND, detection of concentrations down to 100 nM DA is possible. Also, by utilizing surface oxidizing treatments such as nitric acid treatments [18], improvements in the sensitivity have been achieved [5, 7, 20, 22]. Despite all these efforts, the ability to selectively detect DA and AA has not been reached using only metal-free (or very low metal concentration) carbon-based nanomaterials.

**Fig. 4:**
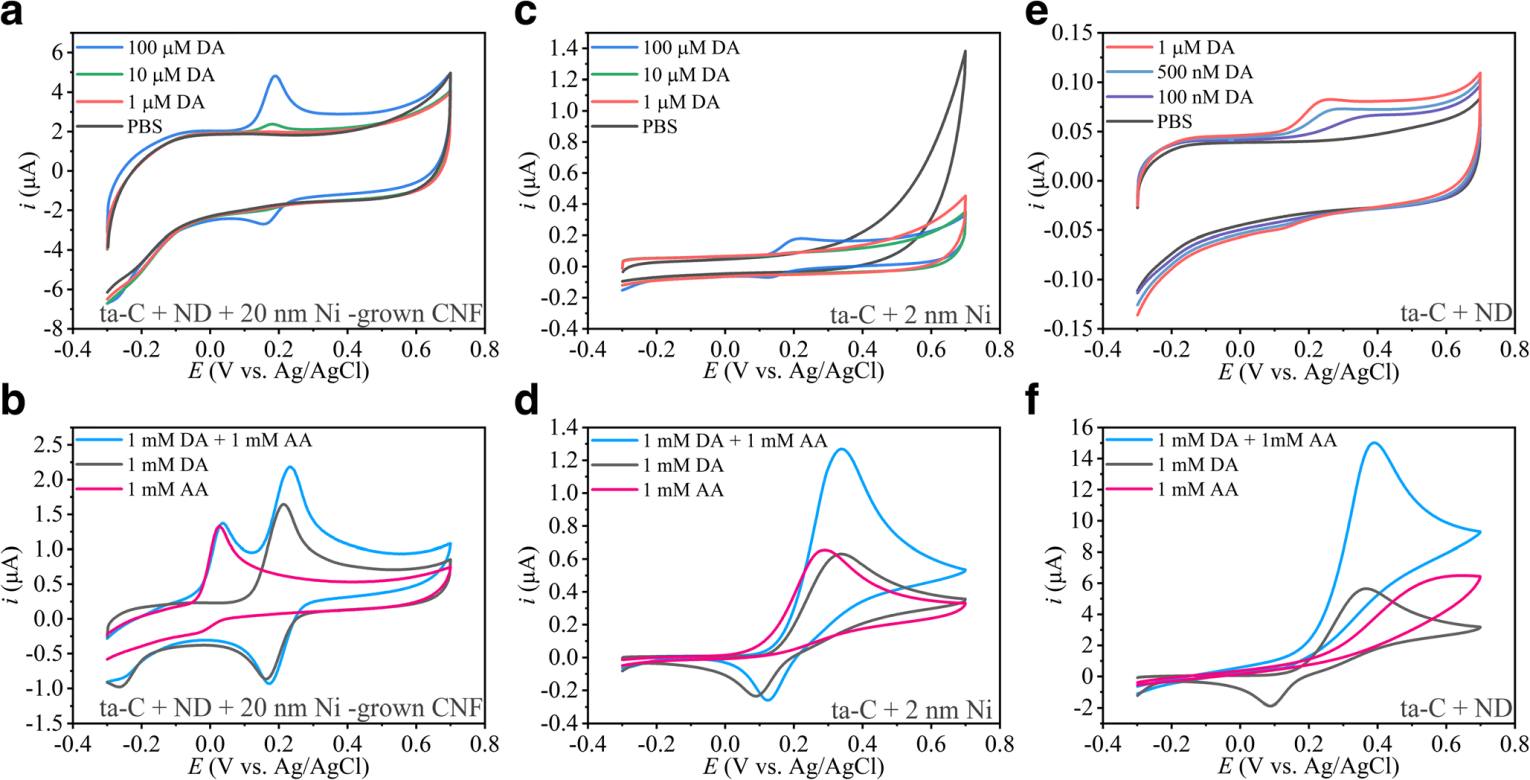
ta-C + ND + 20 nm Ni-grown CNF (**a**, **b**) shows clear selectivity between DA and AA, but only 10-μM detection limit for DA. ta-C + 2 nm Ni in panels **c** and **d** show no selectivity and 100-μM detection limit for DA. In panels **e** and **f**, the ta-C + ND results are shown that show adequate sensitivity but poor selectivity. Scan rate 50 mV/S for all measurements is shown

**Fig. 5:**
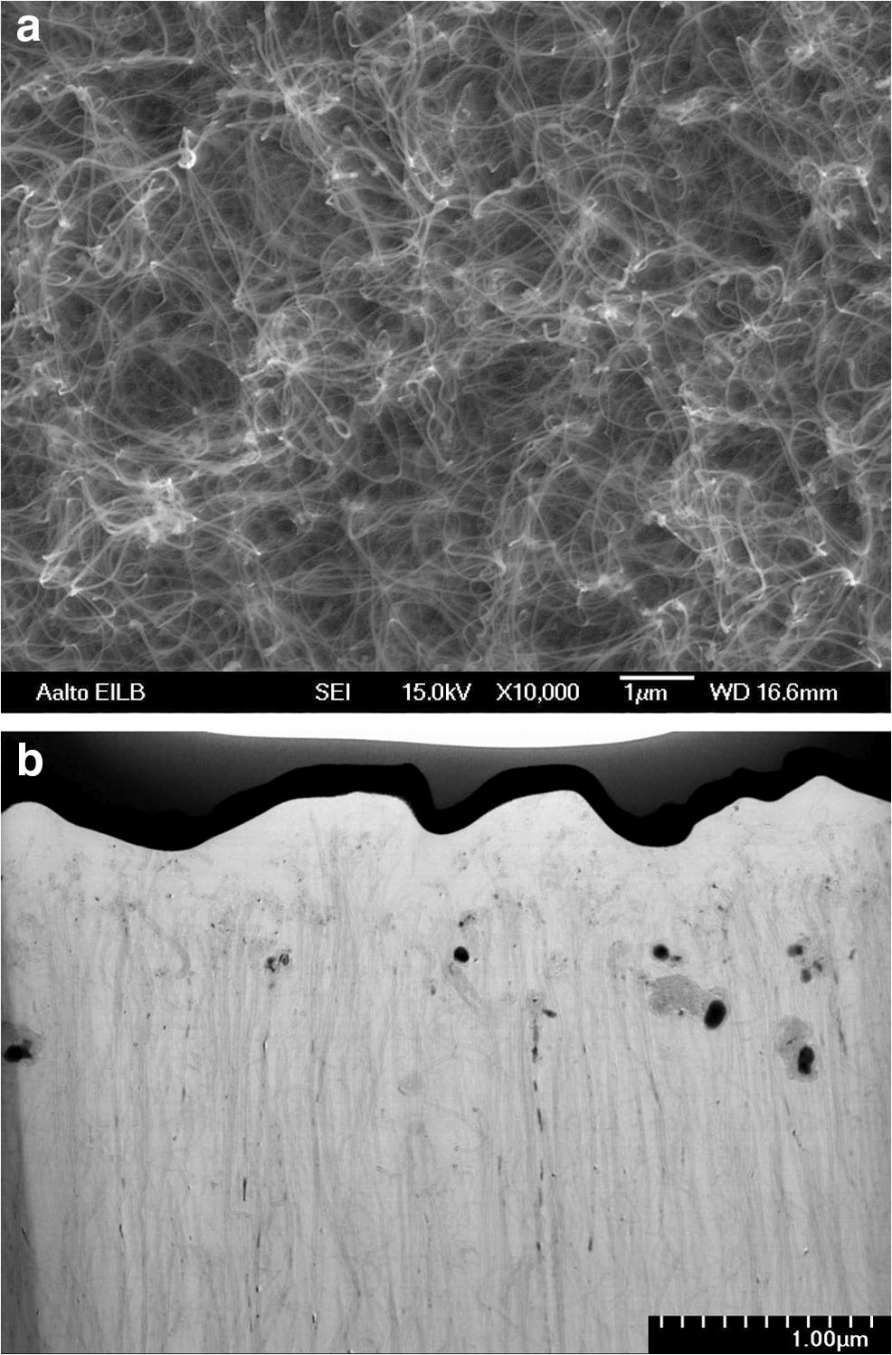
SEM (**a**) and TEM (**b**) micrographs from hybrid CNM with multi-metal seed showing the porous network in panel **a** and the varying sizes and availability of the metal seed particles in panel **b**

As shown earlier, the Ni-grown CNFs are both sensitive for DA and selective between DA and AA [13]. To study the effects of metal and carbon systems in more detail, we introduced an additional 20-nm layer of Ni on top of a ta-C + ND surface before the growth process. The resulting CNF shows selectivity for DA over AA, but the sensitivity for DA was still only 10 μM. This could be related to the fiber structure as its sidewalls, the base, and the tip were different than those for CNF grown without the ND layer (see Fig. 5 in the “Discussion” section for illustration of the different fiber morphologies). Further, we tested deposition of a 2 nm (non-uniform coating) of Ni on top of a 7-nm ta-C. The selectivity for DA and AA was not achieved with this configuration. Thus, it can be concluded that the planar electrode with some Ni particles on the surface did not seem to improve selectivity. Additionally, introducing the Ni on top of the ta-C decreased the sensitivity of the material. Furthermore, a sample with ND deposited on top of the ta-C without growth was measured to inspect its performance. The sample exhibited no selectivity but was sensitive to low concentrations of DA (see Fig. 3e below). These results indicate that the micro- and nanoscale morphology of the CNM is important and the placement of the metal seed particle in the CNM matrix is also relevant. Results from selectivity and sensitivity measurements for the materials listed above are shown in Fig. 4.

To improve the readability and comparability of the results along with laying a clear foundation for the upcoming discussion, we have summarized the results for the studied CNM in Table 2. Table 2 also presents the EDS results from the materials used in this study to confirm the presence of the used catalyst metals (and the Ti adhesion layer used below the ta-C). Correlation of specific amounts of these metals is not discussed in detail as we only know the bulk composition of the materials from the EDS studies. In order to understand the electrocatalytic properties of the materials, both the bulk (such as EDS and fluorescence) and surface characterization (such as X-ray photoelectron spectroscopy and X-ray absorption spectroscopy) should be carried out. The catalyst metal reported in Table 2 has been confirmed using EDS with SEM and/or TEM from the final structure.

**Table 2:** Performance of the different CNM and their physical properties

Geometry and sample type	Metal catalyst	Resulting nanoparticle size	Sensitivity DA	Selectivity DA/AA	Ti adhesion layer exposed	Adequate adhesion between CNM and substrate	Resistance to biofouling	Repeatable performance	Morphology of the CNM	Ti (at%) (*N* = 5)	Fe (at.%) (*N* = 5)	Ni (at.%) (*N* = 5)	Pt (at.%) (*N* = 5)	Co (at.%) (*N* = 5)
Fiber	N/A (ND)		–	–	✓	✓	?	✓	a-C + ND irregular mix	0.08 ± 0.07				
(ta-C + ND)														
Fiber	Fe	80–220 nm	–	✓	–	✓	?	✓	?	0.37 ± 0.02	0.35 ± 0.01			
(ta-C + Fe)														
Fiber	Ni	20–400 nm	✓	✓	–	✓	✓	✓	Herringbone/platelet graphene	0.27 ± 0.04		0.65 ± 0.03		
(ta-C + Ni)														
Fiber	ND + Ni	20–170 nm	–	✓	–	✓	?	?	?	0.16 ± 0.03		0.04 ± 0.01		
(ta-C + ND + Ni)														
Fiber	Pt	10–100 nm	–	–	✓	✓	–	✓	Crystalline, curved graphene	0.25 ± 0.04			0.22 ± 0.02	
(ta-C + Pt)														
Planar	N/A (ta-C)	N/A	✓	–	–	✓	✓	✓	Amorphous	0.22 ± 0.02				
(ta-C)														
Planar	N/A (ta-C + ND)	5–20 nm ND	✓	–	–	–	–	–	Amorphous (ta-C) + crystalline (ND)	0.26 ± 0.03				
(ta-C+ND)														
Planar	ta-C + Ni (2 nm)	< 2 nm	–	–	–	✓	?	✓	Amorphous	0.27 ± 0.01		0.08 ± 0.01		
(ta-C + Ni)														
MWCNT/fiber	Fe + Co + Al	10–65 nm	✓	✓	–	✓	✓	✓	Defective, curved, amorphous + crystalline	0.39 ± 0.02	0.08 ± 0.02			0.23 ± 0.02
(ta-C + Fe + Co + Al)														

### Electrochemical Detection of H_2_O_2_

We have aimed our studies also towards the detection of glutamate (Glu). However, Glu is electrochemically inactive and it is necessary to use enzymes, typically glutamate oxidase (GluOx), and measure the product of the enzymatic reaction. For GluOx, the reaction with Glu in the presence of O_2_ produces H_2_O_2_, which can be detected electrochemically. Thus, materials used for glutamate sensor applications need to fulfill the following criteria: (i) they have to provide a suitable platform for GluOx immobilization and (ii) they have to exhibit good properties for detecting H_2_O_2_. So far, we have inspected H_2_O_2_ detection on several materials including ta-C, ta-C with Pt alloying, ND-grown CNFs, Ni-grown CNFs, and Pt-grown CNFs [6, 19, 23–25]. The best results from these measurements were achieved by using Pt-grown CNFs. We have attributed this in particular for the capability of detecting H_2_O_2_ with the highly catalytic Pt particles at the tip and sidewalls of the fibers. Moreover, we have shown that it is possible to immobilize GluOx on both Ni- and Pt-grown CNFs by covalently cross-linking the enzyme on the carboxylic groups present in the fiber sidewalls and detect Glu with these biosensors [6, 24].

### Electrochemical Detection of Analgesics

We have recently shown that detection of PA and MO simultaneously is possible with planar tetrahedral amorphous carbon (ta-C) thin film electrode if its Ti adhesion layer is partially exposed [7]. Selective detection of PA and MO is not achieved without exposing the Ti and is not possible with just plain Ti.

On the other hand, detecting TR and ODMT sensitively and selectively is only enabled by using ta-C thin films without the underlying Ti. The simultaneous detection is, for the most part, enabled by the large water window of ta-C, allowing separate signals for these two analytes within the measurement window. If a Ti layer is added below the ta-C film, the water window is decreased to an extent where the signal for ODMT is buried under the oxygen evolution. These two examples again emphasize the interplay between chemistry and morphology.

## Discussion

There are several variables that do change between the given CNM that radically change their electrochemical behavior. We can establish the factors that affect the performance include at least (i) the selected metal present in the carbon material, such as the catalyst particles or partly exposed metal sublayer in planar carbon matrix [7]; (ii) the morphology of the surface (porous spaghetti-like, forest-like, planar, fiber width and length and the size of the catalyst particle); and (iii) the nanoscale orientation of the carbon at the surface of the electrode (crystalline or amorphous and the orientation of the graphene planes) and the oxidation state of the carbon and the metal particles on the material surface.

Best results that we have achieved towards neurotransmitter detection are with materials that have (i) the metal seed particles or (ii) are heavily oxidized (and possibly other factors related to listed impurities as stated earlier with the PRGO results) and (iii) have porous-like membrane structures (as the PRGO-layer or CNT layer) that possibly leads to thin liquid film formation that, in turn, promotes adsorption. For H_2_O_2_ detection, we have seen the best results with Pt-grown CNF, which is not surprising owing to the excellent catalytic properties of Pt towards H_2_O_2_. In the detection of MO and PA, the exposure of metal particles is similarly crucial for the separation of the two signals. Contrarily, with TR and ODMT, simultaneous detection is achieved with a ta-C film without an underlying Ti layer and thus without any exposed metal particles [8]. The detection of these was not achieved with Fe-containing SWCNTs.

Looking back to the earlier results, the following obvious conclusion arises: every target application has its own requirements and characteristics and the sensing materials used for one is unlikely to be the best for the other. Thus, understanding the connections between the surface chemistry, nanoscale morphology, and the electrocatalytic behavior is the key to enable fabrication of application-specific CNMs.

For DA, AA, and UA, the best material so far for simultaneous and sensitive detection is the CNM resulting from CVD growth with hybrid Fe + Co + Al seed at low temperature. This material has multi-metal seed particles and a defective porous network of CNM at the surface (see Fig. 5). Similar performance was reached with Ni-grown CNF; however, selectivity was achieved only via DPV [21].

CNFs that show selectivity for DA and AA were grown from both Ni and Fe catalysts, but the latter had too low sensitivity towards DA. Interestingly, the same low sensitivity is also observed for CNFs grown from ND + 20 nm Ni seed layer. Likewise, Ni particles deposited on top of ta-C film showed no selectivity and low sensitivity. This is interesting as it seems that the Ni alone will not solve the selectivity and sensitivity issues as one could falsely conclude from comparing the results obtained with CNF grown from Ni seed and those obtained with ND-grown CNF. Thus, also the intrinsic “nanolevel” morphology of the CNF must be taken into account.

**Fig. 6:**
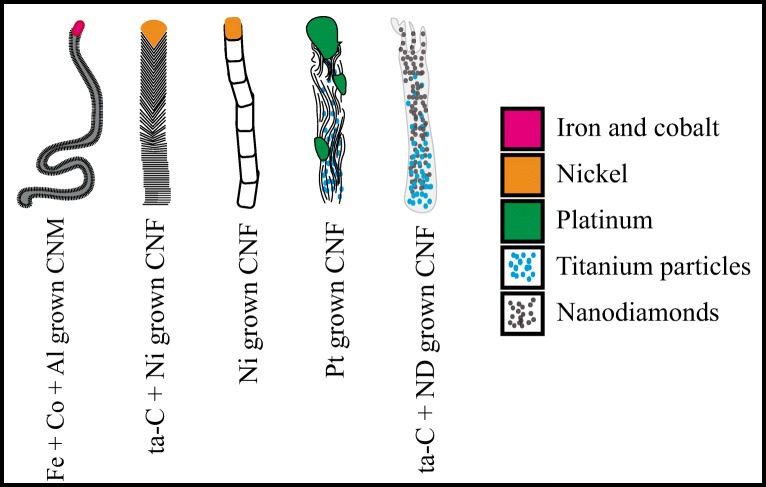
Graphical illustration of the different CNM morphologies

Another aspect may be the size of the nanoparticles residing mainly at the fiber tips. An example is the case with ta-C + 2 nm Ni deposited on top of it. There, the Ni particle size is expected to be small (diameter of just several nm) compared to the variety of particle sizes available in the Ni-grown CNFs which ranges from tens of nanometers in diameter to at least 200 × 400 nm particles [14] (see Fig. 6 for the graphical illustration of the fibers). We have shown that the Ni particles at the fiber tips are oxidized (although not completely) already after the growth process and can be further oxidized at least by acid treatments [18]. Thus, it is expected that polarizing them in electrochemical cell allows subsequent oxidation and reduction of the metal particles promoting electrocatalysis. Further, it is possible that smaller Ni particles would have different electrocatalytic properties than their larger counterparts owing to the more extensive oxidation, for instance. Based on the ta-C + ND + 20 nm Ni-grown CNF, it seems that they have the selectivity as the ta-C + Ni-grown fibers but lack the sensitivity. Here, we expect that the lack of sensitivity is related to the micro- and nanoscale morphology of the fiber. With platelet-type fibers (see references [14, 17, 18], for discussion of the fiber structures), there are edge planes of graphene sheets pointing outwards of the fiber center. This is the case also with other very sensitive fibers [21]. As discussed in the above two references, the sidewalls of the graphene point outside of the fiber center in all cases. Commonalities for both include the following: (i) their structure is crystalline and not amorphous, (ii) there is a plethora of large (tens to hundreds of nanometers in diameter) Ni particles at the tips of the fibers, and (iii) the fibers are protruding from the surface in a forest-like manner.

We anticipate that after further investigation, the ta-C + ND + 20 nm Ni-grown fibers will result in structures of amorphous nature either due to mixing of the ND in the growth process or due to the Ti film reacting differently to the growth process when the ND layer is present and resulting in mixing into the fiber body. The presence of the crystalline ND at the fiber surface could explain why the sensitivity of the ta-C + ND is better than the ta-C electrode alone owing to the increased active area due to roughness.

Figure 6 illustrates the different morphologies of the grown CNF structures and shows the graphical representation of the Ti layer mixing it to the fiber bodies in the ND- and Pt-grown CNF.

In our studies, the Pt-grown CNF foul very quickly (in a matter of few cycles) when used for DA measurements, but are capable of detecting H_2_O_2_ in low concentrations, where the Ni-grown CNF can detect H_2_O_2_ but are less sensitive towards it, and finally, the ND-grown CNFs are the worst of the three for the H_2_O_2_ detection. In comparison, the Fe + Co + Al-grown CNM seems resistant to fouling and the Ni-grown CNF has been found to be resistant as well.

To further improve the performance of the material, as shown earlier, we can enrich the analytes in question by introducing a defective CNM that forms a porous network near the surface.

Interestingly, the ND-grown CNFs are not sensitive towards AA at all (as seen from Fig. 3b). The exact reason for this remains currently unknown. We anticipate that the Ti underlayer, which has been exposed and partially mixed to the fiber body, has a role in the inability to detect AA. If this is truly the case, it is possible that mixing Ti to the common CNM CVD process would allow selective filtering of AA. However, more studies need to be done to prove such hypothesis. These further studies will also include the use of the ND-grown CNF towards detection of PA and MO as the ta-C with the Ti underlayer was capable of detecting them selectively. We envision that the reasons for the inability of AA detection are at least partially related to negative surface charge of TiO_*x*_ (the Ti layer is expected to be completely covered by an oxide) and the ND. This could be true especially with the Vox brand name diamonds, which are sold having a negative zeta potential, if any of the surface functional groups stay intact through the PECVD process. In the cases where Ti is exposed and/or the ND negative zeta potential is making the surface charge of the structure to be negative, there could be an electrostatic repulsion between the CNM and AA (which is negatively charged).

As discussed above, the use of computational methods augmented with machine learning methods can provide us atomic level understanding of the various interactions occurring in the system under investigation and thus provide a tool to tailor surfaces for specific applications. After deconvoluting the experimental XAS spectra, for instance, into fingerprints of various functional groups, we can use this information in our adsorption calculations to mimic the real surface and obtain consequently quantitative understanding of the most feasible functional groups needed for optimal interaction between our target molecule and the electrode surface. We can also use computational screening to obtain information about the most promising catalyst metals for certain types of carbonaceous nanostructures. Although many modern techniques, such as in situ electrochemical STM and AFM, provide us with almost atomic resolution data, its interpretation requires microscopic models, which can be provided only by computational methods. This work is currently underway in our laboratory and several key results in understanding carbon surface chemistry have been achieved [10, 11, 26–28].

## Conclusions

Understanding the root causes of electrocatalytic performance of the carbon-based materials enables their application-specific tailoring. Through machine learning accelerated processing and simulation, a significant amount of time can be cut from the traditional trial-and-error approach in developing novel materials for sensing applications.

Results of our work show that best sensitivity and selectivity towards neurotransmitter DA and its relevant interferents AA and UA was achieved by cyclic voltammetry using hybrid CNM grown from multi-metal seed consisting of Co, Fe, and Al. This material combines the key elements seemingly required for achieving both high sensitivity and selectivity which are (i) active metal catalyst particles present in the CNM structure, (ii) CNM surface has crystalline areas available (basal plane with plenty of defects), and (iii) surface morphology of the material provides increased surface area by forming a porous network possibly resulting into the formation of thin liquid layer near the surface. There are likely numerous other metal-carbon combinations that can achieve similar performance and combining computational methods with the empirical data offers a promising tool for finding them.

We demonstrated that sensitivity towards DA can be achieved with planar, unmodified ta-C surface, and further improved by introducing ND on the surface. These planar electrodes are, however, not selective towards DA and AA. It seems that metal catalyst (Fe, Ni, Fe + Co + Al) at the tips of the CNM or other non-carbon species in the structure (in the PRGO case heavy surface functionalization and/or presence of other elements) are required for selectivity. However, the metal particles alone are not enough to enable the required selectivity. The particles need to be (i) positioned at the tips, sidewalls, or mixed in the CNM matrix; (ii) of certain type(s) for DA detection and DA–AA separation (i.e., Ni or Fe + Co + Al); and (iii) of right size range.

On the other hand, for H_2_O_2_ detection, we found out that the best results were achieved using Pt-grown CNF, whereas Ni and ND-grown CNF can also be used but produce less-viable results. With the Pt-grown CNFs, it is possible to detect H_2_O_2_ at a resolution comparable to pure Pt electrodes with just a fraction of the metal needed. Another advantage of the CNFs over bulk Pt electrodes is the possibility to covalently bind enzymes on them via cross-linking.

For the detection of analgesics, the conclusions are less straightforward. In the case of PA and MO, the best results have been achieved by a ta-C electrode with a Ti underlayer partially exposed. On the other hand, with TR and ODMT, selective detection was enabled by the wide water window of a ta-C electrode with no exposed metal particles.

To conclude, it is evident that the metal particles have a definite role in determining the electrocatalytic properties of the sensing material. It is extremely important to understand how the different metals affect the detection of certain analytes. This could further enable the selection of the desired properties for the given application based on the carbon matrix and morphology. Finally, we emphasize that supporting experimental work with computational methods paired with machine learning offers very fruitful avenues towards solving the unresolved enigma of electrocatalysis.
